# Editorial for Special Issue Photosynthetic Microorganisms: Culturing and Biotechnological Applications

**DOI:** 10.3390/microorganisms13030591

**Published:** 2025-03-04

**Authors:** Eleftherios Touloupakis, Cecilia Faraloni

**Affiliations:** 1 Istituto di Ricerca Sugli Ecosistemi Terrestri, Consiglio Nazionale Delle Ricerche, Via Madonna del Piano 10, Sesto Fiorentino, 50019 Firenze, Italy; 2 Istituto per la Bioeconomia, Consiglio Nazionale Delle Ricerche, Via Madonna del Piano 10, Sesto Fiorentino, 50019 Firenze, Italy; cecilia.faraloni@cnr.it

Microalgae, cyanobacteria, and purple bacteria are photosynthetic microorganisms that are used in many areas, e.g., in agriculture, aquaculture, wastewater treatment, bioremediation, the production of biomaterials, and the generation of renewable energy. They are fast-growing microorganisms that achieve high biomass productivity, mainly use sunlight as an energy source, and have minimal nutrient requirements. The commercialization of photosynthetic microorganisms as a raw material for natural products and biofuels requires the use of efficient cultivation systems. These microorganisms can be grown using a variety of methods, including open and closed systems. Closed photoreactors are the most popular systems, as they allow for optimal growth and reduce the risk of contamination. This Special Issue focuses on the growth and biotechnological applications of photosynthetic microorganisms. Six papers were selected for publication. All six are original studies in which cultivation methods for photosynthetic microorganisms and their biotechnological application are proposed [1,2,3,4,5,6].

In the paper “Blue and Yellow Light Induce Changes in Biochemical Composition and Ultrastructure of *Limnospira fusiformis* (Cyanoprokaryota)”, Pelagatti et al. investigated the effects of different wavelengths of light on the growth of *Limnospira fusiformis* [1]. The aim of the study was to determine whether different types of illumination can cause changes in the storage material in *Spirulina* cells, with a focus on functional microorganisms. The study also aimed to identify specific wavelengths that can cause changes in the chemical composition of *Spirulina*’s biomass, particularly by increasing the total amount of pigments or proteins.

Chacon-Aparicio et al., in the paper “Achieving Discharge Limits in Single-Stage Domestic Wastewater Treatment by Combining Urban Waste Sources and Phototrophic Mixed Cultures”, investigated the potential of co-treatment of domestic wastewater and a liquid stream derived from thermal hydrolysis of the organic fraction of municipal solid waste, mediated by a mixed culture of purple phototrophic bacteria [2]. The aim of the study was to determine the treatment conditions for the recovery of COD, N, and P from domestic wastewater in single-stage open reactors that were enriched with phototrophic purple bacteria.

In the paper “Comparative Analysis of Laboratory-Based and Spectroscopic Methods Used to Estimate the Algal Density of *Chlorella vulgaris*”, Fekete et al. investigated a simple, rapid, and not-resource-intensive estimation method for determining the algal density of *Chlorella vulgaris* based on the measured parameters using UV–Vis spectrophotometry [3]. They developed a general formula for estimating the biomass concentration and gave recommendations for suitable measuring devices based on the algal concentration.

Liu et al. hypothesized in their article “*Symbiodiniaceae* and *Ruegeria* sp. Co-Cultivation to Enhance Nutrient Exchanges in Coral Holobiont” that there is an internal symbiotic loop of *Symbiodiniaceae* and bacteria within the coral symbiotic loop [4]. They conducted experiments to demonstrate how the metabolic exchange between *Symbiodiniaceae* and bacteria facilitates the necessary nutrient supply for cell growth. The study of *Symbiodiniaceae* loop interactions confirmed their hypothesis, which contributes to a comprehensive understanding of the intricate coral holobiont.

Faraloni et al. reported in the article “Dark Anaerobic Conditions Induce a Fast Induction of the Xanthophyll Cycle in *Chlamydomonas reinhardtii* When Exposed to High Light” that *Chlamydomonas reinhardtii* cultures that were incubated under prolonged dark anaerobic conditions showed a stronger induction of the xanthophyll cycle than the dark aerobic cultures when subsequently exposed to high light [5]. These results provide new information on the importance of the redox signaling pathway and highlight the importance of the reductive conditions of the plastoquinone pool in regulating the physiological responses of photosynthetic organisms to stress.

In the article “Mixotrophy in Marine Microalgae to Enhance Their Bioactivity”, Licata et al. investigated the potential applications of certain microalgae species such as *Nannochloropsis granulata*, *Phaeodactylum tricornutum*, and *Chlorella* sp. under different cultivation methods, including phototrophy and mixotrophy [6]. Their study highlights the value of mixotrophic cultivation for increasing the productivity and bioactivity of microalgae, positioning them as versatile organisms for sustainable biotechnological applications.
